# A 57 years old male with giant retroperitoneal mass, lymphocytosis and anemia

**DOI:** 10.11604/pamj.2021.38.413.27670

**Published:** 2021-04-30

**Authors:** Danilo Coco, Silvana Leanza

**Affiliations:** 1 Department of General Surgery, Ospedali Riuniti Marche Nord, Pesaro, Italy,; 2 Department of General Surgery, Carlo Urbani Hospital, Jesi, Ancona, Italy

**Keywords:** Giant retroperitoneal mass, lymphocytosis, anemia

## Image in medicine

Retroperitoneal tumors give non-specific symptoms until they have reached a substantial size. Hystopathological types are: sarcomas such as liposarcoma (70%) and leiomyosarcoma (15%). Other retroperitoneal neoplasms include primary lymphoproliferative tumours (Hodgkin's and non-Hodgkin lymphoma) and epithelial tumours (renal, adrenal and pancreas) or metastatic disease. A 57-year-old male was referred to the emergency department with acute onset of severe melena, right back pain and lipothymia. He had no medical history. On physical examination arterial blood pressure was 70/60mmHg, pulse rate was 100b/min, oxygen saturation of 90%. Blood exams demonstrated lymphocytes 61 x 10^3^/mmc, hemoglobin 6g/dl. Physical examination of the abdomen revealed a giant non-tender mass in the right quadrant. The patient was transfused with 4 units of blood. Abdomen computed tomography (CT) scan revealed a retroperitoneal mass of 20cm with calcification involving the liver, the kidney and surrounding the vena cava. He was referred to a multi-disciplinary team (MDT) to perform a CT scan fine-needle-aspiration (FNA) with a differential diagnosis of lymphoma, sarcoma or renal neoplasia.

**Figure 1:**
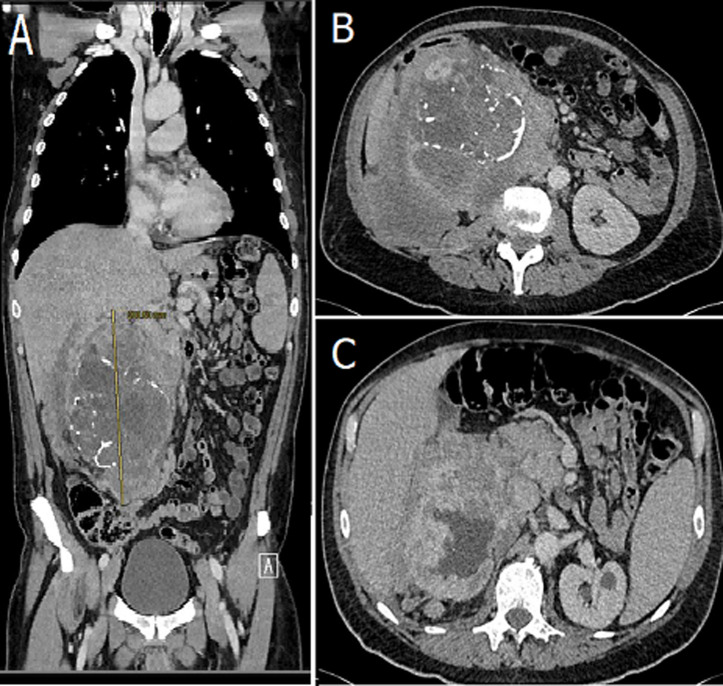
A) abdomen CT scan revealed a retroperitoneal mass of 20cm; B) abdomen CT scan revealed a retroperitoneal mass of 20cm with calcification involving the liver; C) abdomen CT scan revealed a retroperitoneal mass of 20cm with calcification involving the liver, the kidney and surrounding the vena cava

